# Generation of BSA-capsaicin Nanoparticles and Their Hormesis Effect on the *Rhodotorula mucilaginosa* Yeast

**DOI:** 10.3390/molecules24152800

**Published:** 2019-08-01

**Authors:** Alejandro Sánchez-Arreguin, Ramón Carriles, Neftalí Ochoa-Alejo, Mercedes G. López, Lino Sánchez-Segura

**Affiliations:** 1 Departamento de Ingeniería Genética, Centro de Investigación y de Estudios Avanzados del Instituto Politécnico Nacional, Unidad Irapuato, Guanajuato 36824, Mexico; 2 Centro de Investigaciones en Óptica A.C., León, Guanajuato 37150, Mexico; 3 Departamento de Biotecnología y Bioquímica, Centro de Investigación y de Estudios Avanzados del Instituto Politécnico Nacional, Unidad Irapuato, Guanajuato 36824, Mexico

**Keywords:** capsaicin, bovine serum albumin, nanoparticles, fungi, *Rhodoturola*, hormesis

## Abstract

Capsaicin is a chemical compound found in pungent chili peppers (*Capsicum* spp.). In biotechnology, capsaicin has been proposed as a pathogen control; however, its low solubility in water and high instability limits its uses. The aim of this work was to study the effect of high concentrations of capsaicin on the synthesis of nanoparticles and to evaluate their inhibitory effect on the growth of *Rhodotorula mucilaginosa* yeast. Bovine serum albumin (BSA)-capsaicin nanoparticles were formulated at 0, 16.2, 32.5, 48.7 and 65.0 µg of capsaicin per mg of BSA. Nanoparticle properties were evaluated and they were added to cultures of *R. mucilaginosa* to quantify their effect on cell viability. We found that increased capsaicin levels caused several changes to the physicochemical parameters, probably due to changes in the hydrophobicity sites of the albumin during the nanostructuration. The administration of nanoparticles to cultures of *R. mucilaginosa* produced a maximal viability with nanoparticles at 16.2 µg/mg; on the contrary, nanoparticles at 65.0 µg/mg caused maximal cell death. *R. mucilaginosa* cells displayed a hormesis effect in response to the nanoparticle dose concentration. The nanoparticles showed different responses during the uptake process, probably as a consequence of the nanostructural properties of capsaicin in the BSA molecules.

## 1. Introduction 

Plant metabolites represent a group of bioactive compounds, some of which compounds have potential antibacterial and antifungal activity, used as drugs and drug prototypes. Capsaicin [(*E*)-*N*-(4-hydroxy-3-methoxyphenyl)methyl-8-methylnon-6-enamide] is the main chemical compound found in pungent chili peppers (*Capsicum* spp.) [1]. Capsaicin has been applied as a spicy flavouring agent in foods, pharmaceutical treatments and for the control of phytopathogens in plant biotechnology [2,3,4]. Several fungal species showed inhibited germination or decreased growth rates after exposure to capsaicin-methanol or capsaicin-ethanol solutions; *Colletotrichum capsici* exhibited inhibition of conidial germination at 100 and 200 mg/L [2]. However, the uses of capsaicin are limited by its low solubility in water and high instability when exposed to environmental conditions such as light and high temperatures [5]. 

The encapsulation of capsaicin has been proposed as a mechanism to prevent its degradation in industrial applications. The synthesis of nanoparticles containing capsaicin has also been limited due to the materials used and chemical method of assembly. Wang et al. [6] developed microcapsules of polylactic acid (PLA) containing capsaicin prepared by the oil-in-water (O/W) diphase emulsion method. Similar PLA microencapsulates were prepared by the solvent evaporation method, showing its potential use as a biodegradable polymer for controlled release of capsaicin [7]. In previous studies, Sánchez-Segura et al. [8] proposed the use of bovine serum albumin BSA to entrap capsaicin, and they found the optimal conditions to synthesize microparticles; moreover, they observed a tendency of reduction in particle size. Subsequently, Sánchez-Segura et al. [9] found that the concentration of capsaicin used for the elaboration of nanoparticles affected their size, morphology, ζ-potential and other properties. However, the entrapped capsaicin in nanoparticles showed a linear tendency to increase as a function of the capsaicin concentration. Therefore, they proposed that probably there is a conjugation-adsorption limit of capsaicin in the molecules of BSA.

Although, the desolvation-pH-coacervation method is the most studied process in the field of nanoencapsulation [10], the nanostructure of the hydrophilic biopolymer material and the interaction with hydrophobic drugs have not been completely explained. On the contrary, it has been simplified as two steps: 1) accumulation of the desolvating agent produces an increment of particle size, and 2) a step where the particle size remains constant, but the particle concentration increases [11]. Sánchez-Segura et al. [9] found that capsaicin produces changes in the secondary structure of BSA during the elaboration of BSA nanoparticles, allowing the modification of its hydrophobic regions. The modification of these regions produces a new quaternary assembly that promotes the capsaicin entrapment process confining the hydrophobic drug to the core of the resulting nanoparticles. Subsequently, the nanoparticles are stabilized through a process of fusion between nanoparticles. As mentioned above, it is probable that the BSA molecules show a large capacity for entrapment of capsaicin and the nanoparticles allow an increased bioactive potential effect of capsaicin.

The fungus *Rhodotorula mucilaginosa* is a member of the phylum Basidiomycota which has a wide ecological distribution and can be found in air, soil, high altitude lakes, ocean water, hypersaline water and high-temperature environments [12]. The ecological niches of *R. mucilaginosa* include colonization of plants and animals as natural hosts. Moreover, products commonly consumed or used by humans like foods, medical instruments, shower curtains, bathtubs and toothbrushes very often show the presence of this microorganism. Some *R. mucilaginosa* strains are considered non-virulent to humans, however, in recent reports an unusual pathogenicity in HIV patients, in chronic diseases such as renal failure, lymphoproliferative disease, organ transplantation, broad spectrum therapy and invasive medical procedures has been described [13]. Moreover, the ubiquitous and fast proliferation of this fungus has become a public health issue [14]. Kurita et al. [15] studied the mechanisms of antimicrobial activity of capsaicin in *Saccharomyces cerevisiae* and found that capsaicin treatment at 250 ppm induced 39 genes from approximately 5560 and 121 genes were repressed, several of which are essential for cell viability. For this reason, the aims of this work were to study the effect of high concentrations of capsaicin in the fabrication of nanoparticles, and also to analyse the effect of these particles on the growth of *Rhodotorula mucilaginosa*.

## 2. Results and Discussion 

### 2.1. Yield and Efficiency of the BSA-Capsaicin Nanoparticles 

**Figure 1:**
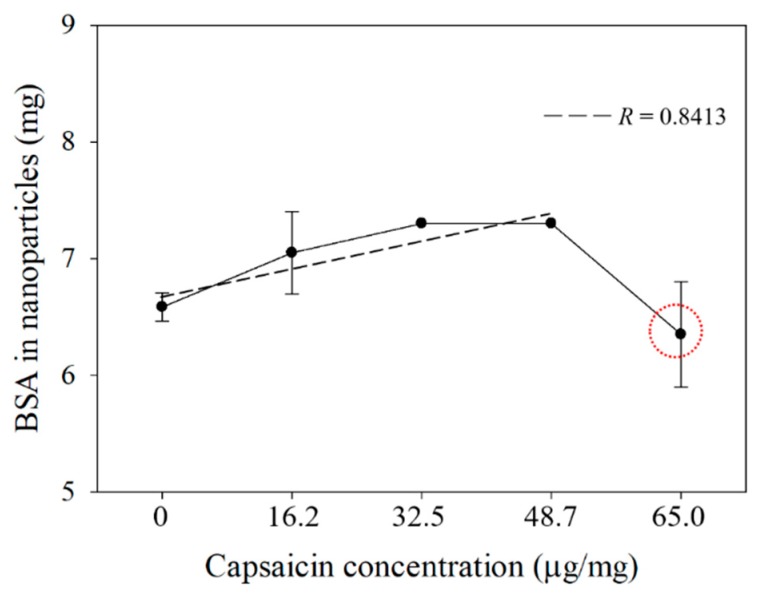
BSA protein transformed into nanoparticles as a function of capsaicin concentration. The quantified protein showed a linear tendency (R = 0.8413) in relation to the concentration. Values are means of three experimental replicates ± standard deviations.

**Figure 2:**
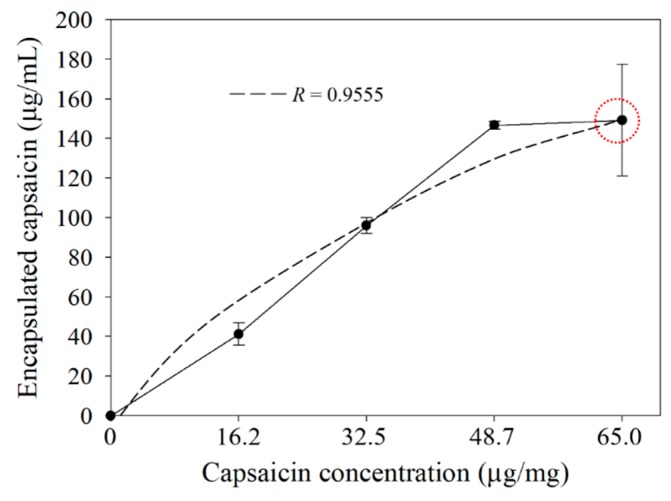
Encapsulated capsaicin in nanoparticles with respect to the concentration. The quantified capsaicin showed a logarithmic tendency (R = 0.9555). Values are means of three experimental replicates ± standard deviations.

In treatments at 0, 16.2, 32.5 and 48.7 µg/mg of capsaicin per mg of BSA, an increment of the quantified BSA was observed. The BSA protein that was transformed into nanoparticles showed a linear tendency (R = 0.8413) in relation to the concentration of capsaicin (Figure 1). Nevertheless, at 65.0 μg/mL of capsaicin, the BSA concentration was decreased. Similar behaviour was observed for nanoparticle yield; the highest value was above 80% at 32.5 and 48.7 µg/mg. However, at 65.0 µg/mg the yield decreased (Table 1). This phenomenon observed at the highest concentration of capsaicin was probably due to the reorganization of the BSA molecules during the final step of the desolvation process, whereby the nanoparticles were disassembled and transformed in soluble BSA during the crosslinking step; such a process was not reported in the previous work by Sánchez-Segura et al. [8,9]. On the other hand, a logarithmical tendency (R = 0.9555) was found in the quantification of encapsulated capsaicin (Figure 2). This result showed that the gradual increment of capsaicin in the desolvation solution, produced high interactions between BSA and capsaicin molecules. The exposure of BSA molecules to ethanol-capsaicin solution probably produced sequential changes in the native BSA (albumin N isoform) structure, changing it from an α-helix to a β-sheet form (albumin F isoform); under these conditions the hydrophobic regions of the BSA were probably exposed and the capsaicin showed affinity to the amino acids tyrosine, asparagine, lysine, phenylalanine, histidine, methionine and leucine; subsequently, the complex albumin F isoform-capsaicin probably recovered the α-helix structure as described by Baler et al. [16], during the final step of desolvation the BSA-capsaicin complex acquired the nanoparticle shape. However, at 65.0 μg/mg of capsaicin, a more hydrophobic environment was achieved in the coacervation solution and the saturation of the hydrophobic regions of BSA was probably reached, resulting in disassembly of the BSA nanoparticles (Figure 1, circle), probably freeing the excess capsaicin entrapped in the hydrophobic regions of BSA (Figure 2, circle). 

A similar effect was observed in the encapsulation efficiency (EE%) of the capsaicin in the nanoparticles (Figure 2). The EE% showed a typical increase for treatments from 16.2 to 48.7 µg/mg of encapsulated capsaicin. The efficiency parameter at 48.7 µg/mg showed the major quantity of entrapped capsaicin (72.2%) with a similar quantity of BSA transformed into nanoparticles (80.3%) with respect to the 32.5 µg/mg of capsaicin concentration. However, the lowest EE% was observed at 65.0 µg/mg (Table 1); this effect was probably due to the saturation of the hydrophobic sites of BSA with capsaicin, as mentioned above. A similar effect of the concentration of capsaicin was observed by Sánchez-Segura et al. [9]; they found that an increase in the capsaicin concentration affected both the yield of BSA nanoparticles, and the efficiency of the drug entrapped in the nanoparticles due to an increased exposure of the hydrophobic sites in the albumin resulting in a major affinity for hydrophobic drugs. De Freitas et al. [17] reported that the rate of ethanol addition (1 mL/min) had a higher impact on EE%; they attributed this effect to the process of drug encapsulation which possibly requires lower rate during the desolvation stage. However, they did not put forth a possible mechanism and the effect of the rate on the formation of nanoparticles from the albumin-capsaicin complex. It is also probable that the yield and efficiency parameters might be affected by the molecular crystallinity of the encapsulated drug. The transition stage of F-albumin allows the encapsulation of the ethanol-capsaicin complex in solution. However, recovery of the *N*-albumin shape produces reorganization of the BSA molecules, forming an external wall of nanoparticles and an internal core of crystalline capsaicin. A similar effect was observed by Luebbert et al. [18] during a study on the formation of protein aggregates and increased size of dodecanoic acid-defatted BSA nanoparticles; they found that the secondary structure of BSA protein did not show changes during the fatty acid loading procedure. However, the loading efficiency (similar to EE%) and the diameter of nanoparticles were affected; they proposed a reorganization of the fatty acid in the nanoparticles as a possible mechanism that explains these changes. This phenomenon was confirmed by Bhalekar et al. [19]; they found that the transition stages of crystallinity of lipid drugs affected the assembly of nanoparticles as well as the EE%.

### 2.2. Structural Changes of BSA-Capsaicin Nanoparticles Characterized by FTIR

**Figure 3:**
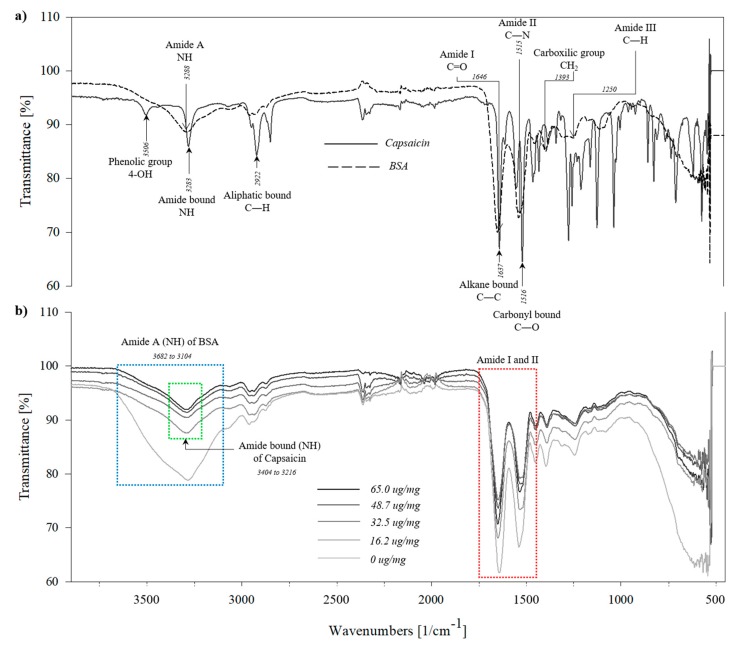
FTIR characterization of the BSA–capsaicin pure molecules and the effect of the increment of capsaicin in the nanostructure formation. (**a**) FTIR spectral characterization of BSA and capsaicin; the scanning spectral range was between 650–4000 cm^−1^. (**b**) Spectral graphs of the BSA-capsaicin nanoparticles at 0, 16.4, 32.5, 48.7 and 65.0 µg/mg, (lines in gradient of grey). The treatments showed between 3682 to 3104 cm^−1^ a deformation of the peak that corresponds to N–H stretching (rectangle blue), and the second region is the amide I and amide II of the BSA with the hydrophobic side chain of the capsaicin, from 1710–1126 cm^−1^.

The assignment of the vibrations of the functional groups of capsaicin molecules can be interpreted in different ways. In previous reports, the FTIR spectra of capsaicin were reported to show three characteristic peaks at 3278, 2922 and 1600 cm^−1^ [20,21]. However, we found slight changes with respect to the previous report because the first peak was observed at 3506 cm^−1^, the second at 3283 cm^−1^, the third at 2922 cm^−1^ and finally, a group of peaks was seen from 1637 cm^−1^ to 1516 cm^−1^. For this study, a satisfactory interpretation considered the fundamental modes of vibration by observing the position, shape and intensity of bands [22]. The first peak was assigned to –OH groups; this functional group has been described at 3700–3300 cm^−1^ for plant metabolites, and in the capsaicin this resonance probably corresponds to the phenolic 4-OH group [23], detected at 3506 cm^−1^ (Figure 3a). The second peak corresponds to the amide bond N–H stretching of the capsaicin molecule [24]; this functional group has been described at 3450–3250 cm^−1^. The third peak was attributed to the aliphatic C–H bond stretching vibration; this functional group is usually found in the spectral range from 3100–3000 cm^−1^ [22]. However, for the capsaicin molecule, the bands were recorded at 2927 cm^−1^ and 2874 cm^−1^. Finally, the resonances at 1626, 1596, 1553 and 1528 cm^−1^ were assigned to the C–C and C–O stretching vibrations; the frequency showed a range from 1600–1528 cm^−1^ (Figure 3a, solid line) [22]. On the other hand, the BSA native protein spectrum showed a major peak at 3288 cm^−1^ that corresponds to amide **A**, related to N–H stretching, at 1646 cm^−1^ the vibrance corresponds to amide **I** associated to C=O stretching, at 1515 cm^−1^ the peak was attributed to amide **II** related to C–N stretching and N–H bending vibrations. The CH_2_ bending groups were associated to the peak at 1393 cm^−1^. The vibration of the peak at 1250 cm^−1^ corresponds to amide **III** related to C–N stretching and N–H bending (Figure 3a, dotted line); a similar spectrum was reported by Bronze-Uhle et al. [25].

On the other hand, the BSA nanoparticles with 0 µg/mg and BSA-capsaicin nanoparticles with 16.2, 32.5, 48.7 and 65.0 µg/mg of capsaicin concentration showed a deformation of the peak that corresponds to the N–H amide **A** stretching of BSA (Figure 3b, blue rectangle). We found that in the region between 3682 to 3104 cm^−1^, the structure of the BSA nanoparticles showed a predominance with respect to the phenolic 4-OH group and NH amide bond of capsaicin (Figure 3b, green rectangle). However, in the treatment with 65.0 µg/mg an increase of the NH amide bond signal of capsaicin (N–H stretching) in the region from 3404 to 3216 cm^−1^ (similar to the peak of pure capsaicin mentioned above), respect to the amide **A** of BSA was observed; this change was probably due to the reorganization of the amino acid residues on the surface of BSA to the centre of the nanoparticle structure and to the exposure of capsaicin on the surface of the nanoparticles. The second region (1703–1494 cm^−1^) with a high interaction corresponds to the amide **I** and amide **II** bands of BSA with the hydrophobic side chain of the capsaicin (Figure 3b, red rectangle). The FTIR results of BSA-capsaicin nanoparticles are completely different when compared with the more recent report of De Freitas et al. [17] on the interpretation of the FTIR analysis about the association of the functional groups of capsaicin and BSA molecules in a different context. This interpretation did not explain the relationship of BSA-capsaicin nanostructuration and simplified the interaction through the hydroxyl groups of capsaicin with the sulfhydryl groups of cysteine of BSA, due to the large quantities of BSA molecules found. Anand et al. [26] described 14 interactions in the capsaicin−BSA complex (Lys535− capsaicin C15, Phe550−capsaicin C15, AHis534−capsaicin C16, Met547−capsaicin, Leu582−capsaicin C15, Lys524−capsaicin, Leu531−capsaicin C15, BHis534−capsaicin C15, Leu528−capsaicin) of which five (Tyr400−capsaicin O2, Asn401−capsaicin H49, Lys524−capsaicin H34, Phe506−capsaicin H27, and Phe550−capsaicin H36) are considered strong interactions, mostly involving hydrophobic and electrostatic interactions, and none of these interactions is with cysteine.

### 2.3. Morphology of Nanoparticles

**Figure 4:**
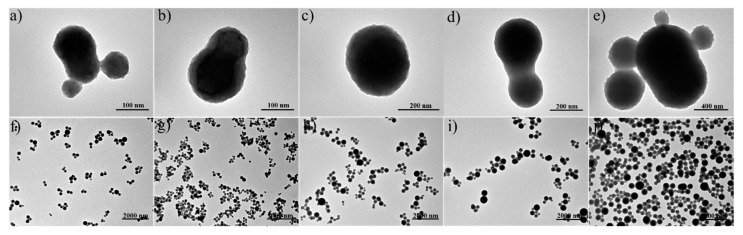
TEM micrographs at high magnification show isolated nanoparticles with details in the morphology and nanostructure. TEM micrographs at low magnification show size and shape distribution of BSA-capsaicin nanoparticles at different capsaicin concentrations. All experiments in TEM were carried out at 80 kV high voltage (EHT) at 710,000× to 140,000× (high magnification), and at 8900× to 9000× (low magnification), working pressure 5 × 10^−3^ Pa (5 × 10^−5^ Torr).

TEM micrographs of the nanoparticles showed a morphological change as a result of the increase in capsaicin concentration. The nanoparticles at 0 µg/mg of capsaicin mainly exhibited particles with an elliptical shape and secondary particles branching the structure (Figure 4a, the image was captured at 110Kx) at low magnification (8900×); the distribution of the particles showed a similar shape with a variable size (Figure 4f). The nanoparticles at 16.2 µg/mg showed a similar main particle without fused secondary particles; the surface of the particles was slightly rough (Figure 4b, the image was captured at 110Kx) at low magnification (8900×), the distribution of the particles presented similar morphology and size (Figure 4g). Subsequently, at 32.5 µg/mg, it was possible to observe that the particles became circular in shape and showed a reduction of size (Figure 4c, the image was captured at 140Kx); moreover, at low magnification (9000×), the distribution of the particles exhibited homogeneity in shape and size (Figure 4h). 

Nanoparticles in the treatment with 48.7 µg/mg of capsaicin showed larger particle sizes, probably due to their coalescence (Figure 4d, image was captured at 71Kx). Observations at low magnification (8900×) allowed the visualization of the columnar structures of nanoparticles with an increment in heterogeneity (Figure 4i). Finally, the morphology of nanoparticles at 65.0 µg/mg of capsaicin showed a loss of circularity, probably due to coalescence and fusion between nanoparticles of high size (high mass) and small particles. At this stage, the nanoparticles showed an electrodense area where the core previously was; the intense staining with uranyl acetate indicated the great presence of free amino –NH and –COOH groups in the nanoparticles [27]. Moreover, the surface of the nanoparticles was rougher and wrinkled respect to other treatments (Figure 4e, the image was captured at 56Kx) possibly because the capsaicin had moved from the core to the surface of the nanoparticles. At low magnification (8900×), the particles showed aggregations in the form of columns and round structures (Figure 4j). The variability of morphological and morphometrical features can be attributed to the high degree of hydrophobicity of the encapsulated drug [9]. In the nanoencapsulation of hydrophilic drugs (5-fluorouracil, vinorelbine tartrate and salicylic acid), drastic changes of the physicochemical properties were not observed [25,28,29]. 

### 2.4. Effective Diameter (Ed), Aspect Ratio (Ar) and Shape Factor (Sf) of the Nanoparticles 

**Figure 5:**
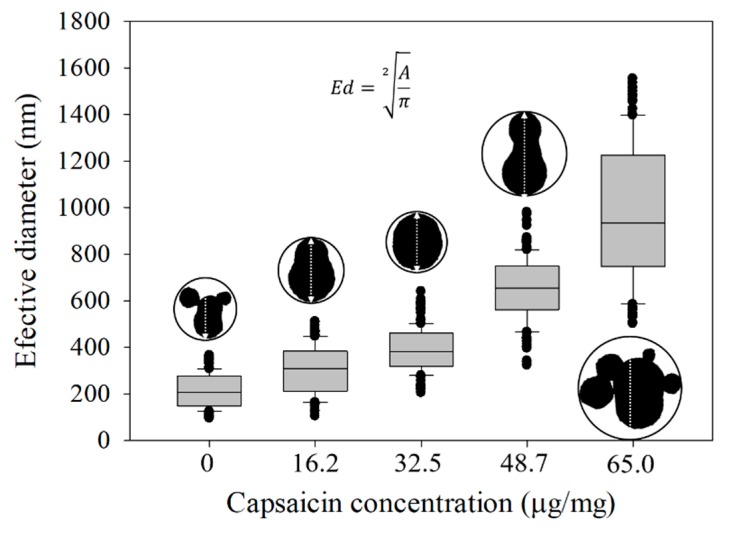
Effective diameter of the isolated nanoparticles showed an increasing tendency with respect to the concentration of capsaicin. The equation of effective diameter (*Ed*) is also displayed. Conditions: digital measurement of *n* = 180 nanoparticle images at 1376 × 1032 pixels and 8 bits of compression; resolution set at 2.1 pixels/nm. Values are the means of three experimental replicates ± standard deviations.

Morphometric parameters were calculated by image analysis process from images of nanoparticles acquired by TEM. The *Ed*, *Ar*, and *Sf* parameters describe the size, shape, and circularity of nanoparticles. They all showed a change in function of the increment of capsaicin concentration. An increase on particle diameter was observed between all treatments without capsaicin (0 µg/mg) and with capsaicin (16.2, 32.5, 48.7 and 65.0 µg/mg), the average *Ed* was 212, 304, 390, 652 and 981 nm for the corresponding treatments with increasing capsaicin concentration, respectively (Figure 5). 

Moreover, this parameter showed an increment of dispersion values, indicating that changes in the size of the nanoparticles were probably due to coalescence between nanoparticles, forming aggregates themselves. A similar response was observed by Sánchez-Segura et al. [9]; the authors demonstrated that the increment of capsaicin concentration produced a change on the shape of nanoparticles by increasing the coalescence events between nanoparticles. The coalescence was probably due to an increase of the hydrophobicity into the BSA molecules by exposure of their hydrophobic area, this process increases the instability between BSA molecules. The increment and/or decrement of the particle size is consistently observed in interactions between materials of different degree of hydrophobicity. Choi et al. [30] found that particles elaborated with *Capsicum* oleoresin and several different wall materials exhibited fluctuations in the diameter size of the nanoparticles. Luebbert et al. [18] found that native BSA (with fatty acids) showed an increase in nanoparticle sizes with respect to fatty acid-free BSA (DF-BSA). However, the diameter of particles showed a maximal increment of diameter respect to the concentration of the dodecanoic acid (12 C in length) added during the nanostructuration process. This result coincides with our findings.

**Figure 6:**
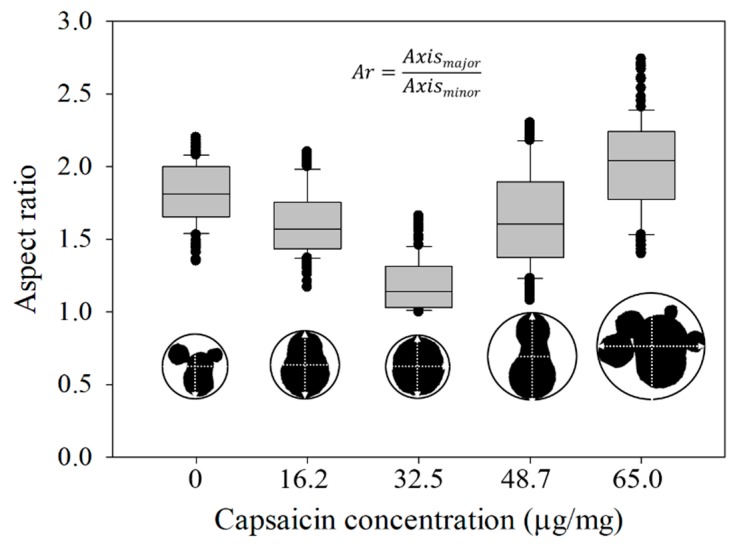
Aspect ratio of the isolated nanoparticles showed a gradual transition from irregular dimension (X-Y axis) to circular shape and irregular aspect at high concentration of capsaicin. The equation of aspect ratio (*Ar*) is also displayed. Conditions: digital measurement of *n* = 180 nanoparticle images at 1376 × 1032 pixels and 8 bits of compression; resolution set at 2.1 pixels/nm. Values are the means of three experimental replicates ± standard deviations.

**Figure 7:**
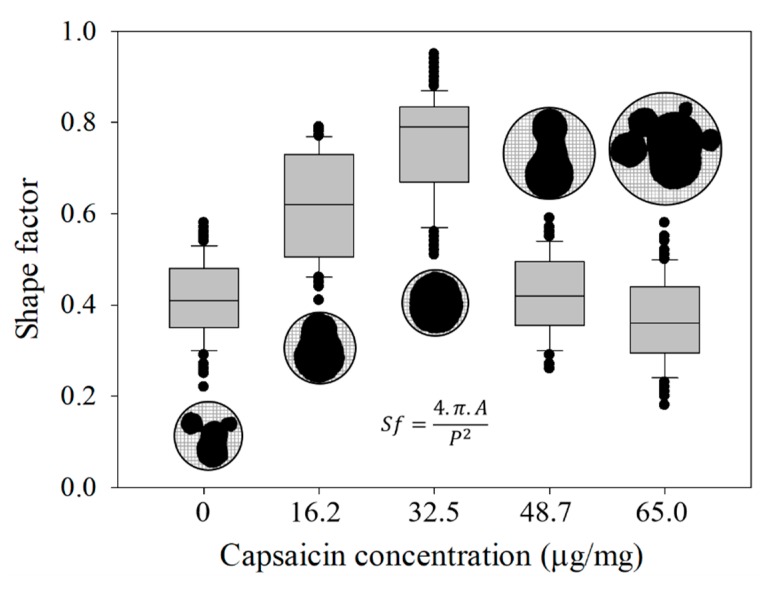
Shape factor of the isolated nanoparticles showed the best circular shape at 32.5 μg/mg of capsaicin concentration. Subsequently, the increase in capsaicin concentration caused a loss of shape. The equation of shape factor (*Sf*) is also displayed. Conditions: digital measurement of *n* = 180 nanoparticle images at 1376 × 1032 pixels and 8 bits of compression; resolution set at 2.1 pixels/nm. Values are the means of three experimental replicates ± standard deviations.

On the other hand, the aspect ratio (*Ar*) and shape factor (*Sf*) showed an asymmetrical response. The values for the capsaicin concentrations 0, 16.2, 32.5, 48.7 and 65.0 µg/mg were *Ar =* 1.81, 1.62, 1.19, 1.65 and 2.02, while *Sf =* 0.41, 0.61, 0.76, 0.42 and 0.37, respectively (Figure 6 and Figure 7). 

This means that the nanoparticles showed the most circular shape at 32.5 µg/mg. However, the treatment without capsaicin (0 µg/mg) and the treatments with higher capsaicin concentrations (65.0 µg/mg), showed similar shapes (elliptical and irregular morphology). Moreover, it is important to mention that in these treatments a low dispersion of the values was observed; this suggests that probably the aggregations with columnar and round structures are more stable, and on the contrary, nanoparticles with circular shape are considered less stable. At high capsaicin concentration (65.0 µg/mg), the loss of circular shape was probably due to the coalescence of nanoparticles due to the formation of capsaicin crystals, affecting the coagulation of the BSA molecules. In a parallel way, the BSA showed a change of electrical charge, thus altering their electrostatic properties [31]. However, in the 0 µg/mg capsaicin treatment the nanoparticles did not show similar features of *Ar* and *Sf* parameters with respect to those of Sánchez-Segura et al. [9]. This effect was probably due to the change of stirring speed (1100 rpm) used during the coacervation process. 

### 2.5. ζ-Potential, Polydispersity Index (PDI) and Hydrodynamic Diameter of Aggregates

The ζ-potential showed an increment of the nanoparticles electronegative charge at low and medium concentrations of capsaicin. At 0 µg/mg of capsaicin, the electrical charge was −45.51 ± 1.31 mV, while it was −48.26 ± 1.72 mV and −50.09 ± 1.18 mV for 16.2 µg/mg and 32.5 µg/mg of capsaicin, respectively, showing a decrease of the ζ-potential. We observed the lowest ζ-potential at 48.7 µg/mg showing −54.82 ± 3.75 mV. However, at 65.0 µg/mg an increase was observed in the ζ-potential (−47.18 ± 1.22 mV) (Supplementary Figure S1). Statistical analysis showed a significant difference (*p* < 0.05) between 0 and 16.2 µg/mg and treatments with 48.7 µg/mg and 65.0 µg/mg of capsaicin. Li et al. [32], found in native BSA that the a zeta potential increase correlated negatively with the hydrophobicity as a function of decreasing pH from 7.0 to 3.0; the positive charge of the BSA surface was associated to its unfolded structure, i.e., the acid amino acid residues were reorganized on the BSA molecular surface while the basic ones were distributed inside the BSA molecule. A similar effect can be attributed to the increment of capsaicin (16.2 µg/mg, 32.5 µg/mg and 48.7 µg/mg), the hydrophobic environment probably produces a change of pH into the nanoparticles producing more exposition of the basic amino acid in the nanoparticles surface. In an opposite way, at 65.0 µg/mg treatment, the saturation of capsaicin produces a similar effect to that reported by Li et al. [32] and the coalescence of the nanoparticles occurred by unfolding of the BSA structure. It is possible that the cause of the electric charge alteration on the nanoparticles surface may be due to changes in the amino acids exposed on the nanoparticle surface.

On the other hand, the mean value of the aggregate size and PDI showed an oscillatory trend in relation to the concentration of capsaicin. At 0 μg/mg of capsaicin, the average diameter of aggregates was 252.42 ± 41.00 nm and PDI 0.38 ± 0.06; the aggregate size increased to 314.96 ± 34.41 nm at 16.4 μg/mg, and the PDI increased to 0.39 ± 0.06. At 32.5 μg/mg a decrease of the aggregate size to 327.43 ± 43.25 nm and an increase of PDI to 0.23 ± 0.04 were observed. At 48.7 μg/mg of capsaicin, the aggregate size showed a similar value (327.59 ± 32.46 nm) respect to the previous treatment, and PDI showed a slight increment (0.29 ± 0.04). Finally, at 65.0 μg/mg of capsaicin, the aggregate size and PDI increased (569.28 ± 45.33 nm; 0.98 ± 0.07). The aggregation mean value and PDI of the BSA-capsaicin nanoparticles showed a drastic change at 65.0 μg/mg. As mentioned above, this phenomenon was attributed to an increased hydrophobicity of BSA molecules and a reorganization of the amino acid residues in the nanoparticle structure surface; the reduction of the net charge of BSA molecules produced an unfolding of the BSA structure [32]. Then, the intermolecular electrostatic repulsion improved the possibility of aggregation, this effect is known as coalescence. The coalescence between nanoparticles produced a loss of circularity, aggregation, aberrant morphology and ζ-potential changes [9].

### 2.6. Isolation and Identification of R. mucilaginosa

The amplified DNA fragments obtained from genomic DNA of the *R. mucilaginosa* culture showed an electrophoretic pattern (Supplementary Figure S2). The identity of yeast sequences was determined by comparison with the sequences available in the GeneBank/EMBL/DDBJ (http://www.ncbi.nlm.nih.gov). The combination of band lengths from ITS1 and the ITS4 profiles allowed the identification of *Rhodoturola mucilaginosa* yeast species from the mucilage of a water deposit; a microscopic observation confirmed the presence of unicellular fungi. All sequences were deposited in the GeneBank under their accession numbers: 18S rRNA: SUB5872749 and ITS1-4: SUB5873084 for reference to this manuscript.

### 2.7. Hormesis Effect of NPs BSA-Capsaicin in R. mucilaginosa

**Figure 8:**
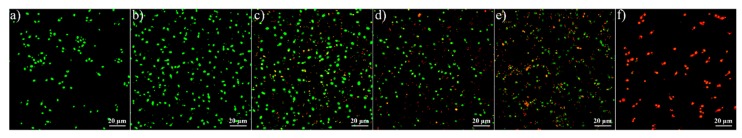
The AO/PI staining shows the live and death cells in all treatments. (**a**) Control treatment with buffer showed a basal growth of *R. mucilaginosa*. (**b**) Treatment with 0 µg/mg shows an increment of the cell population. (**c**) The maximal increment of viable population of *R. mucilaginosa* was observed at 16.2 µg/mg of capsaicin. (**d**) Treatment with 32.5 µg/mg causes a decrease in mature viable cells of *R. mucilaginosa* fluorescent in green channel and an increase in the young death cell in red channel. (**e**) At 48.7 µg/mg, the growth inhibition was observed in the mature cell. (**f**) The mature death cells were observed at 65.0 µg/mg and no young cells were observed.

Hormesis is a term used by toxicologists to refer to a biphasic dose response to an environmental agent characterized by a low dose stimulation or beneficial effect and a high dose inhibitory or toxic effect [33]. The inhibition effect of capsaicin on seed germination and conidial germination has been described in different studies. It is known that the inhibition of germination is dependent on the concentration of capsaicin; a high capsaicin concentration causes greater mortality of cells [2,34]. However, in this study the exposure of *R. mucilaginosa* to BSA-capsaicin nanoparticles, a hormesis behaviour in the fungi cell growth was observed. AO/PI staining allowed us to distinguish between live and dead cells in the treatment cultures. Nevertheless, we added a buffer assay for comparing normal growth of *R. mucilaginosa* with the BSA-capsaicin nanoparticle treatments. In the control (only buffer) an abundant presence of yeasts with green fluorescence was observed (Figure 8a); the yeast culture exhibited mostly viable cells with 63.83 × 10^6^ ± 3.5 × 10^6^ cells/mL and a minor quantity of dead cells (1.86 × 10^6^ ± 0.57 × 10^6^ cells/mL), while in the 0 μg/mg treatment, the population of viable *R. mucilaginosa* was increased 89.6 × 10^6^ ± 1.85 × 10^6^ cells/mL; no inhibition of growth was observed (cell death 0.33 × 10^6^ ± 0.05 × 10^6^ cells/mL), and the images showed abundant yeast fluorescing in the green spectrum (Figure 8b). At 16.2 μg/mg of capsaicin (low concentration), the culture presented an increase in green fluorescent yeasts (123.16 × 10^6^ ± 10.88 × 10^6^ cells/mL) and death cells (2.70 × 10^6^ ± 1.32 × 10^6^ cells/mL) were observed with more frequency in all images (Figure 8c). The tendency to increase the viability of *R. mucilaginosa* changed at 32.5 μg/mg, since the viability was only observed in mature cells 92.73 × 10^6^ ± 2.90 × 10^6^ cells/mL, whilst, death cells increased the population 5.16 × 10^6^ ± 1.80 × 10^6^ cells/mL. Interestingly, many of the dead cells were smaller, which implies that the most susceptible cells to BSA-capsaicin NPs were the youngest cells (Figure 8d), probably due to the permeability of their cell walls. At 48.7 μg/mg of capsaicin (high concentration) a decrease of the viable yeast population (68.70 × 10^6^ ± 5.65 × 10^6^ cells/mL) was seen; the dead cells showed an increased population (42.03 × 10^6^ ± 12.63 × 10^6^ cells/mL). It must be noted that the dead cells were young cells, produced during yeast budding events. However, mature dead cells were also observed (Figure 8e). Finally, at 65.0 μg/mg of capsaicin, the high concentration treatment caused complete growth inhibition; the dead cells showed a major value (75.66 × 10^6^ ± 5.69 × 10^6^ cells/mL), whereas on the contrary, the viability of cells showed the lowest value of all treatments (3.56 × 10^6^ ± 3.20 × 10^6^ cells/mL). Interestingly, the images showed mature dead cells (Figure 8f). 

In this treatment the capsaicin nanoparticles produced growth inhibition by an unknown mechanism. Kurita et al. [15] found in *S. cerevisiae* cultures treated with capsaicin a possible molecular effect on gene repression i.e., *RPS25A*, *RPS29A* and *RPS8A* related to ribosomal components, *DLD1* to mitochondrial oxidoreductase, *HHT2*, *HTA1* and *HHF1* to cellular growth and essential genes of the cellular biogenesis and protein synthesis. However, an alternative effect may be the atrophy of the mechanism of multi-drug resistance membrane transporters (superfamily genes *PDR*-type ABC).

**Figure 9:**
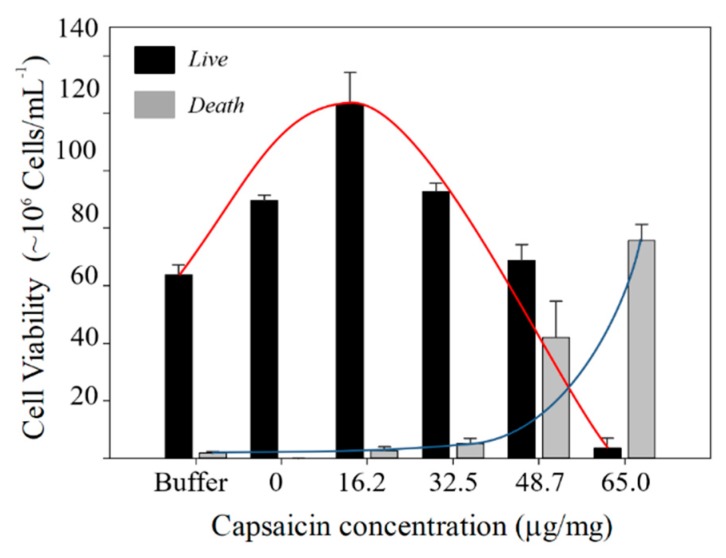
Hormesis response of *R. mucilaginosa* cells in the presence of BSA-capsaicin nanoparticles administrated directly to the culture. The live cells showed maximal growth at 16.2 µg/mg and growth inhibition at 65.0 µg/mg (the red line shows the positive hormesis behaviour). The death cells showed minimal activity at 0 µg/mg and maximal activity of nanoparticles at 65.0 µg/mg (the blue line shows the negative hormesis behaviour).

The growth of the yeast showed higher stimulation than the controls at low capsaicin concentration (16.2 μg/mg), followed by inhibition at the highest capsaicin concentration (65.0 μg/mg), this phenomenon is considered as a hormesis effect; the graph of the viability counterstain showed a typical dose-response effect resembling an inverted U-shape graph for a positive effect and J-shape graph for a negative effect (Figure 9). 

### 2.8. Cellular Uptake and Release of Capsaicin

**Figure 10:**
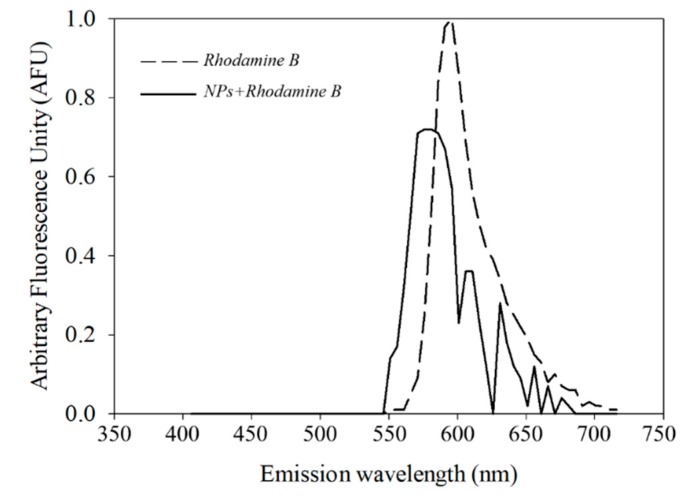
Comparative spectra of fluorescence emission of rhodamine B staining. (**a**) Rhodamine B diluted in water at 1% showed a maximal peak emission at 600 nm. (**b**) Nanoparticles of BSA-capsaicin stained with rhodamine B showed a maximal peak emission at 575 nm.

In order to verify the nanoparticle cellular uptake, the nanoparticles were stained and characterized as described in the Methods section. The spectral characterization of the rhodamine B solution showed an emission between 560 to 710 nm, showing a maximal emission peak at 600 nm, while the BSA-capsaicin nanoparticles exhibited an emission between 540 to 690 nm, showing a maximal peak at 575 nm (Figure 10). 

This result suggests that rhodamine B is an effective fluorophore to track the NP uptake; moreover, the fluorescence of rhodamine B showed a compatibility with calcofluor white for counterstain the cell wall of yeasts. 

**Figure 11:**
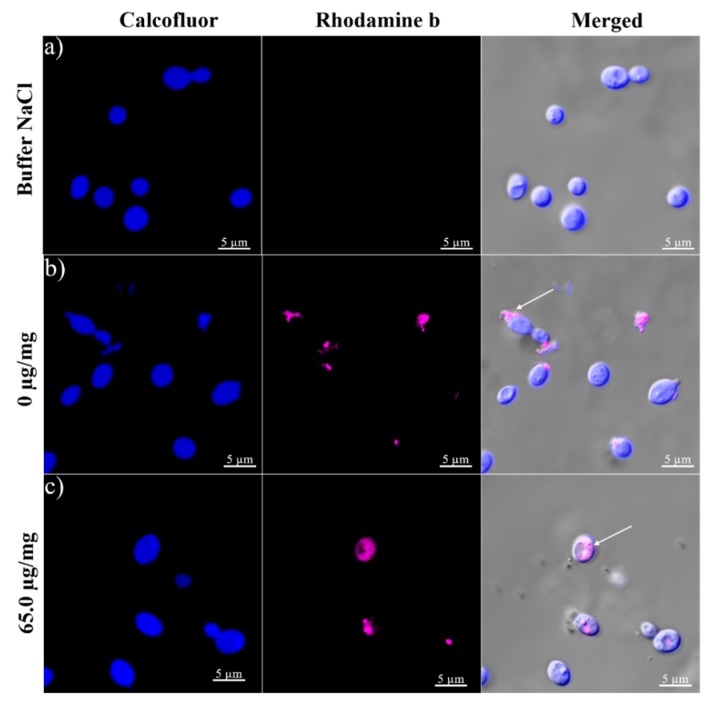
BSA-capsaicin nanoparticle uptake by cultures of *R. mucilaginosa*. (**a**) Treatment with NaCl buffer did not show rhodamine B signal. (**b**) Treatment at 0 µg/mg showed nanoparticles aggregated on the surface of cells (see white arrow). (**c**) Accumulation of the BSA-capsaicin nanoparticles staining with rhodamine B into the *R. mucilaginosa* cell was observed at 65.0 μg/mg.

In this experiment, we used a buffer control to compare the effect of NPs on the cell wall of *R. mucilaginosa* with respect to those that were not exposed to NPs. After the incubation time yeast cells treated with buffer showed a homogeneous cell wall, and the texture of the surface presented no alterations and no BSA-capsaicin fluorescence was detectable (Figure 11a). Noticeable changes were observed in the treatment with 16.2 μg/mg of capsaicin; the nanoparticles aggregates were bound to the surface of the yeast cells, this mechanism probably produce a certain dosage of nanoparticles (Figure 11b, white arrow). The cell wall of the *R. mucilaginosa* showed no changes in texture or morphology, therefore the internalization of the nanoparticles did not produce changes in the microstructure of the cell wall. An intracellular accumulation of BSA-capsaicin nanoparticles was observed at 65.0 μg/mg; the cytoplasmatic area exhibited rhodamine B fluorescence around the vacuoles (Figure 11c), moreover, the texture of the cell wall showed dark spots, probably caused by damage to or cell wall disassembly. 

Although the nanoparticle treatment with the highest concentration of capsaicin (65.0 μg/mg) showed the lowest % BSA yield (69.8 ± 4.9) and % encapsulation efficiency (55.1 ± 10.4); the % BSA yield and % encapsulation efficiency are not parameters that describe themselves the mechanism of nanostructuration. 

The % BSA yield and % EE only describe the stability of the chemical process during the encapsulation [35]. We found that treatment at the highest concentration of capsaicin (65.0 µg/mg) produced a larger change in the molecular arrangement between BSA protein and capsaicin respect to treatments formulated with lower concentrations of capsaicin. These changes in the molecular arrangement of the nanoparticles are possibly due to a translocation of capsaicin from the core to the surface of the nanoparticles, changing the structure and function of the BSA-capsaicin. The FTIR and ζ-potential measurements showed changes more related to capsaicin molecules signal in the treatment at the highest concentration respect to the other treatments. However, the increment of capsaicin in the formulation produced great events of coalescence; this phenomenon generates a fusion between nanoparticles and the loss of circularity, aggregation, aberrant morphology and changes on the surface of nanoparticles. 

The morphometric parameters most affected were the effective diameter (981 nm) and the size of the aggregates (569.28 ± 45.33 nm), which were found to increase; however, the aggregates are probably formed from single nanoparticles fused by coacervation events (Figure 4j). The internalization of single nanoparticles in *R. mucilaginosa* yeast could be possible by breaking the aggregated nanoparticles with enzymes secreted by the yeast, changing the pH of the culture medium. Single nanoparticles also might be internalized to the cytoplasmic area by simple diffusion through the cell wall and cell membrane (Figure 11c). The inhibitory growth effect showed by BSA-capsaicin nanoparticles formulated at 65.0 µg/mg of capsaicin was probably due to the exposure of capsaicin structure on the surface of the nanoparticles (see Graphical Abstract). The possible mechanism of cell death triggered by the nanoparticles in *R. mucilaginosa* is a matter of further research.

## 3. Materials and Methods

### 3.1. Reagents and Chemicals

The biological reagents used in this study were BSA powder (66430.0 g/mol) (Equitech-Bio, Kerrville, TX, USA) and pharmaceutical grade capsaicin (≥99%) from *Capsicum* spp. (Shanghai, China). The chemical reagents were glutaraldehyde 25% (Electron Microscopy Science, Hatfield, PA, USA) sodium chloride analytical grade (Merck, Darmstandt, Germany), acetonitrile (≥99.9%) (Sigma-Aldrich, St Louis, MS, USA) and absolute ethanol (≥99.8%) (Merck), calcofluor white water solution 1% (Sigma-Aldrich), rhodamine B powder (Hycel, Jalisco, México), orange acridine (Sigma-Aldrich) and propidium iodine (Sigma-Aldrich).

### 3.2. Preparation of BSA-Capsaicin Nanoparticles

Nanoparticles were prepared with a desolvation technique as described by Langer et al. [10] modified by Jahanban-Esfahlan et al. [36] and Sánchez-Segura et al. [9]. Briefly, 200 mg of BSA powder were dissolved in 2 mL of 10 mM NaCl solution, pH 9.4 and filtered through a 0.22 μm syringe filter (Sartorius, Goettingen, Germany). The solution was maintained under agitation at 200 rpm controlled by a laboratory stirrer (Eurostar 20, IKA, Wilmington, NC, USA) for 30 min at room temperature. Coacervation was generated by addition of 4 mL of an ethanol-capsaicin solution at 0, 16.2, 32.5, 48.7 and 65.0 µg/mg of capsaicin (per mg of BSA) for each treatment. The rate of addition was 1.0 mL/min at 1100 rpm of stirring speed. The crosslinking process was carried out by the addition of 5 mL of 4% glutaraldehyde in a 10 mM NaCl solution in agitation at 1000 rpm for 30 min in dark conditions. The particles were washed by three cycles of centrifugation at 12,485× *g* for 10 min at room temperature (MC-12V, DuPont, Newtown, CONN, USA), and dispersion of the pellet was carried out in 10 mM NaCl at pH 9.4, during every cycle. Each dispersion step was performed by shaking in a vortex mixer (Super Mixer, LAB LINE, Melrose Park, ILL, USA) for 10 min. At the final centrifugation cycle, 2 mL of NaCl solution at pH 9.4 were added to prepare the stock. The purified particles were stored at 4 °C.

### 3.3. Determination of ζ-Potential, Polydispersity Index (PDI) and Hydrodynamic Diameter of Aggregates

The ζ-potential, PDI and hydrodynamic diameter of aggregates were determined using dynamic light scattering (Zetasizer Nanoanalyser ZSP, Malvern Instruments, Worcester, U.K.) on mode scattering angle of 12.8°. The zeta potential was evaluated using a 1:20 dilution factor in deionized water. Deionized water allows the measurement of the surface electrical charge of nanoparticles and reduces the NaCl ions in solution. Data were automatically evaluated with the Smoluchowski equation in which particle size of ≈100 nm is much larger than the Debye length, ≈1 nm [37]. Measurements were made in a folded capillary zeta cell (DTS0012, Malvern Instruments). Data of zeta potential were analysed using the SigmaStat software version 3.5.0 (Systat Software Inc, San Jose, CA, USA). The differences in the median values among the treatment groups were carried out by one-way analysis of variance (ANOVA). The multiple comparison procedure to isolate the group or groups that differ from the others was carried out by Tukey method. Significance was considered at *p* ≤ 0.05.

### 3.4. Quantification of BSA Transformed in Nanoparticles and Encapsulated Capsaicin 

The quantification of encapsulated capsaicin in nanoparticles was done by the recovery of capsaicin in acetonitrile [38]. Volumes of 500 μL nanoparticles were washed with deionized water by three cycles of centrifugation at 12,485× *g* for 6 min at room temperature; the supernatant was discarded at the final step. The pellets were lyophilized (HETO MAXY dry, LYO, Lillerød, Denmark), the dried nanoparticles were broken by addition of 1 mL acetonitrile; the samples were homogenised for 10 min (Super Mixer, LAB LINE) and sonicated for 5 min at 30 °C. The samples were centrifuged at 12,485× *g* for 10 min (MC-12V, DuPont). Finally, the supernatants were filtered through an acrodisc of 0.22 µm pore size (Sartorius) and directly deposited in a HPLC vial; the samples were kept at −20 °C. Denaturalized protein was incubated at 30 °C for 1 h in order to evaporate residual acetonitrile. Weights of samples were registered using an analytical balance (Pioneer, Ohaus Analytical Plus, Shanghai, China).

Quantification of capsaicin was performed by HPLC (1290, Agilent, Santa Clara, CA, USA). The separation of capsaicin was achieved with a Zorbax Eclipse Plus C18 column with 130Å pore size, (1.8 μm, 2.1 mm × 50 mm) (Part number: 959757-902, Agilent) and reversed-phase consisting of water (A) and acetonitrile (B) [A:B (40:60, *v*/*v*)] in an isocratic mode at 1.0 mL/min of flow rate. The absorbance of eluted material was monitored at 280 nm and ultraviolet (UV) spectra were recorded in the range of 220–350 nm at an acquisition rate of 1.25 scan/s. The calibration curves were obtained from a capsaicin standard at different concentrations (500, 1000, 5000 and 10,000 µg/mL). The curve and samples were prepared by injecting 15 μL in real triplicates.

BSA nanoparticles yield and encapsulated efficiency were calculated with a modification of the equations described by Bhaleka et al. [19] with slight modifications. The estimation of the BSA transformed into nanoparticles was adjusted to a total volume (11 mL) recovery from the nanoparticle coacervation process. The proposed Equations (1) and (2) were computed for each experiment as follows:

(1) $$BSA\;nanoparticles\;yield\;\left(\%\right)=\frac{\mathrm{BSA}\;\mathrm{in}\;\mathrm{nanoparticle}\;\left(\mathrm{mg}\right)}{\mathrm{initial}\;\mathrm{BSA}\;\left(\mathrm{mg}\right)}\times 100$$

(2) $$Encapsulation\;efficiency\;\left(\%\right)=\frac{\mathrm{encapsulated}\;\mathrm{capsaicin}\;\left(\text{µ}g\right)}{\;\mathrm{initial}\;\mathrm{capsaicin}\;\left(\text{µ}g\right)}\times 100$$

### 3.5. Fourier Transform Infrared Spectroscopy (FTIR) of BSA-Capsaicin Nanoparticles

Fourier transform infrared spectra of pure capsaicin, pure BSA and treatments of BSA-capsaicin nanoparticles were recorded using an FTIR spectrometer (Cary 660, Agilent Technologies, Santa Clara, CA, USA) with attenuated total reflectance (ATR) fit. The configuration used in the equipment was crystal of zinc selenide (ZnSe) for scanning spectral range between 650–4000 cm^−1^ and with open-cell transmission DialPath (optical path lengths 50, 100 and 250 μm) and diamond ATR accessories. Capsaicin standard and BSA samples were homogenised and analysed directly, whereas nanoparticles samples were washed with deionized water by three cycles of centrifugation at 12,485× *g* for 6 min at room temperature; the supernatant was discarded at final step. The pellet was lyophilized (HETO MAXY dry, LYO) for 3 h and subsequently homogenised and analysed. Sixty-four scan were recorded with a nominal resolution of 4 cm^−1^ in transmittance mode [%T]. Single-beam spectra of the samples were collected against a background of air. Three replicates of each sample were averaged to one spectrum. Spectral data were decoded with spectroscopy software SpectraGryph version 1.2 (Dr. Friedrich Menges Software-Entwicklung, Oberstdorf, Germany). The spectral graphs were analysed in Sigma plot 12 (Systat Software, Inc.).

### 3.6. Transmission Electron Microscopy (TEM) and Morphometric Analysis of Nanoparticles

The morphology of the nanoparticles was examined by TEM (Morgagni M-268, Philips/FEI, Brno, Czech Republic). Samples of nanoparticles (5 μL) were placed onto 200 mesh formvar/carbon coated copper grids (Ted Pella Inc., Redding, CA, USA) and incubated for 10 min. Samples were contrasted with 2.5% uranyl acetate (Electron Microscopy Science Inc., Hatfield, PA, USA) and incubated for 15 min. The TEM operating conditions in all experiments were 80 kV high voltage (EHT) from 8900× to 9000× for low magnification (*shadow magnification*) and high magnification from 56,000× to 180,000× at low vacuum pressure 5 × 10^−3^ Pa (5 × 10^−5^ Torr). Micrographs were captured in tagged image file (.tif) format with 1376 × 1032 pixels in grey scale. In this format, 0 was assigned to black and 255 to white in the grey scale.

The images of nanoparticles for each treatment were cropped and transferred into a new image of 1376 × 1032 pixel with format .tif at 8 bits of compression in white background. The resolution of the images was 2.1 pixels/nm. The morphometric descriptor was calculated using the “shape_descriptor1u” plugin for ImageJ v.1.49p software (National Institutes of Health, Bethesda, MD, USA). The parameters of the particles were effective diameter (*Ed*), aspect ratio (*Ar*) and the shape factor (*Sf*). *Ed* calculates the diameter of a fictitious circular object that has the same area as the object being measured according to Syverud et al. [39] (Equation (3)). *Ar* was calculated according to Parakhonskiy et al. [40] and describes the relation between the width and length of the particles. The *Ar* is a suitable description of ellipticity e.g., for “flat” particles *Ar* < 1, for circular particles *Ar* = 1, and for elliptical particles *Ar* > 1 (Equation (4)). *Sf* or circularity is based on the projected area of the particle and the overall perimeter of the projection according to Bouwman et al. [41] (Equation (5)):

(3) $$Ed=\sqrt[{2}]{\frac{A}{\pi }}$$

(4) $$Ar=\frac{Majoraxis}{Minoraxis}$$

(5) $$Sf=\frac{4\cdot \pi \cdot A}{P^{2}}$$

### 3.7. Yeast Sampling and Genetic Identification of R. mucilaginosa

The yeast was isolated from the water deposit of a dehumidifier air device. The yeast inoculum was growth in PDB liquid medium supplemented with 2 µL of carbenicillin antibiotic during 24 h at 28 °C in darkness condition under agitation at 200 rpm. The purity of the cultures was verified by an optical microscope on the basis of their cell morphology. The genetic identification was carried out by genomic DNA from pure cultures of yeast. DNA was isolated using the glass bead lysis method as described by Hoffman and Winston [42]. DNA concentration was measured by its absorbance at 260 nm with a nanodrop spectrophotometer (ND-1000, Thermo Scientific, Waltham, MA, USA) and its integrity observed by electrophoresis on agarose gels. Amplification of 18S rRNA and ITS fragments was carried out in thermal cycler equipment (Gradient, Techne, Staffordshire, U.K.) with the following PCR program: 1 cycle at 94 °C, 3 min; 35 cycles of 94 °C, 30 s; 60 °C, 30 s; and 1 min at 72 °C, and finally 1 cycle at 72 °C for 7 min. Each reaction mixture contained 5 µL of 10× high fidelity PCR buffer, 1 µL of 10 mM dNTP mixture, 2 µL of 50 mM MgSO_4_, 2 µL of 10 µM of primers mix 18S F (5′AAGGGGAATCTGACTGTC 3′) and 18S R (5′CTCATTCCAATTACAAGACC 3′), or 2 µL of 10 µM of primer mix ITS1 (5′TCCGTAGGTGAAC CTGCGG 3′) and ITS4 (5′TCCTCCGCTTATTGATATGC 3′) [43], 50 ng of genomic DNA, 0.2 µL of Platinum Taq High Fidelity (Invitrogen, Waltham, MA, USA) and milli-Q water was added to each tube reaching the final reaction volume of 50 µL.

### 3.8. Nanoparticle Staining with Rhodamine B and Uptake by R. mucilaginosa

To 1 mL of nanoparticles at 0 µg/mg was added 1 μL rhodamine B solution at 1% (Hycel, Jalisco, México) for staining and incubated for 15 min under darkness at room temperature; subsequently, the sample was washed three times with deionized water. The spectrum of emission of the rhodamine B was analysed directly from the stock solution. The samples were mounted on glass slides, covered with high performance Zeiss cover glasses (D = 0.17 mm +/− 0.005 mm refractive index = 1.5255 +/− 0.0015, Abbe number = 56 +/− 2) and observed in a multiphoton microscope system (LSM 880–NLO, Zeiss, Oberkochen, Germany) equipped with an Ti:Sapphire laser (Chameleon vision II, COHERENT, Santa Clara, CA, USA) capable of tuning in ranges from 690 to 1060 nm. Detection of spectral emission of rhodamine B and nanoparticles stained with rhodamine B was performed by multiphoton microscopy. The samples were observed and analysed with immersion objective 60×/1.4, NA ∞−0.17, Zeiss Plan NEOFLUAR. The rhodamine B spectrum (from 547 to 602 nm) was obtained from the data-base of Zeiss microscope dyes. The experimental detection of the spectrum of rhodamine B pure and nanoparticles dyed with rhodamine B was carry out with chameleon laser syntonised at 810 nm with 0.2% of power and detected at 538–656 nm with a photomultiplier GaSP at 900 mV of sensibility. All micrographs were captured in CZI format at 1131 × 1131 pixels and RGB colour. The spectral graphs were analysed in Sigma plot 12 (Systat Software, Inc.). 

### 3.9. Determination of Viability of R. mucilaginosa 

The estimation of *R. mucilaginosa* viability was determined using orange acridine-propidium iodide staining [44]. The initial culture was activated in 20 mL of potato dextrose broth (PDB) liquid medium supplemented with 20 µL of the antibiotic carbenicillin (50 mg/mL) for 24 h at 28 °C in dark conditions under agitation at 200 rpm. Cellular concentration was calculated with a haemocytometer [45]. Subsequently, an aliquot of 20 µL of initial culture with 2,052,333 ± 15,176 cells (*n* = 3) was added to 2 mL of PDB supplemented with 2 µL of carbenicillin antibiotic (50 mg/mL) and 100 µL of nanoparticles (treatments at 0, 16.2, 32.5, 48.7 and 65.0 µg/mg); an additional treatment with NaCl solution pH 7.4 at 10 mM was carried out for contrasting the effect of the nanoparticles with normal growth of *R. mucilaginosa*. The cultures were grown at 28 °C for 24 h in dark conditions with rotation at 10 rpm. Subsequently, 1 mL of culture was aliquoted and washed by three cycles of centrifugation at 6242× *g* for 4 min; the yeast was resuspended in 100 µL of deionized water and then incubated with 50 µL acridine orange (AO) 0.01%—50 µL propidium iodide (PI) 0.002% for 15 min in darkness at 4 °C. The staining process was completed by three cycles of centrifugation at 6242× *g* for 4 min and resuspended at a 1:100 dilution in deionized water; 1 μL of this sample was taken for microscopy. For viability tests, 1 μL was imaged in confocal microscopy system (LSM 880–NLO, Zeiss) with an immersion objective 60×/1.4, NA ∞−0.17, Zeiss Plan NEOFLUAR. The acridine orange was excited at 488 nm and emission was recovered from 504–580 nm and propidium iodide was excited at 543 nm and emission was recovered from 590–730 nm. All samples were captured in 15 micrographs (with three repetitions) at 1.4X-1.4Y of zoom, equivalent to 9,291 µm^2^ of scanned area. All images were captured in .CZI format at 1131 × 1131 pixels and RGB colour. Analysis of live and death yeast cells was carried out with a “cell counter notice” plugin and morphometric description was calculated by “shape_descriptor1u” for ImageJ v.1.49p software (National Institutes of Health).

### 3.10. BSA-Capsaicin Nanoparticles Uptake by R. mucilaginosa and Intracellular Distribution

To freshly cultures of *R. mucilaginosa* 100 µL of BSA-capsaicin nanoparticles previously stained with rhodamine B were added; the treatments evaluated were nanoparticles at 0 and 65.0 µg/mg of capsaicin concentration. An additional treatment with NaCl solution pH 7.4 at 10 mM was added as control of the fluorescence of rhodamine B. The cultures were grown at 28 °C for 24 h in darkness with rotation at 10 rpm. After that time, 1 mL of culture was aliquoted and washed by three cycles of centrifugation at 6242× *g* for 4 min; the yeast was resuspended in 100 µL of deionized water and then incubated with 2 µL calcofluor white water solution 1% for 30 min in darkness at 4 °C. Finally, the samples were washed by three cycles of centrifugation at 6242× *g* for 4 min; the yeast was resuspended in 100 µL of deionized water. Samples were imaged in a multiphoton microscopy system (LSM 880–NLO, Zeiss), the rhodamine B was excited with chameleon laser syntonised at 810 nm with 0.2% of power and emission was recovered from 538-656 nm and calcofluor white was excited at 735 nm with 2.2% and the emission was recovered at 411–496 nm. All micrographs were captured in .CZI format at 1131 × 1131 pixels and RGB colour. 

## 4. Conclusions

The increase of capsaicin concentration in the formulation of nanoparticles possibly affected the structural organization of the hydrophobic sites of BSA molecules providing greater capacity to assimilate capsaicin during the synthesis of nanoparticles. Capsaicin showed double activity, as an encapsulated drug and as an inductor of hydrophobicity in the coacervation process. Under the present conditions, BSA presented a greater capacity to entrap capsaicin compared to previous works [8,9]. However, saturation of the desolvation solution was found at 65.0 μg/mg of capsaicin; at this level, capsaicin probably produces destabilization of the nanoparticle structure and unfolding of the BSA, causing changes on the physicochemical properties of nanoparticles among them and then the release of entrapped capsaicin. The administration of BSA-capsaicin nanoparticles to *R. mucilaginosa* proved that they can be used to inhibit the cell growth of fungi. However, the effect of the encapsulated capsaicin depends on the capsaicin concentration so that the lowest concentration of capsaicin (16.2 µg/mg) stimulates the growth and development of the *R. mucilaginosa* cells. Only at the highest concentration of capsaicin (65.0 μg/mg) did the yeast cultures show a high mortality and growth inhibition rate. *R. mucilaginosa* cells displayed a hormesis effect in response to the concentration of the nanoparticle dose applied. The reported BSA-capsaicin nanoparticles are a nanobiotechnology development that might be a useful tool for pathogen fungi control. 

## Figures and Tables

**Table 1:** Nanoparticle yield and entrapment efficiency of capsaicin.

Capsaicin Concentration (µg/mg)	BSA Nanoparticles Yield (%) ^a^	Encapsulated Efficiency (%) ^b^
0	72.4 ± 1.3	0.0 ± 0.0
16.2	77.5 ± 3.8	60.9 ± 8.3
32.5	80.3 ± 0.0	70.8 ± 2.9
48.7	80.3 ± 0.0	72.2 ± 0.9
65.0	69.8 ± 4.9	55.1 ± 10.4

^a,b^ n = 3 ± SD.

## Supplementary Materials

The supplementary materials are available online. Figure S1: The ζ-potential of BSA-capsaicin nanoparticles showed a decrease in the electronegativity associated to the accumulation of capsaicin into the nanoparticle core. At 65.0 µg/mg the saturation with capsaicin produces a significant increment of the ζ-potential. Figure S2: Electrophoretic patterns of PCR products of the 18S rRNA gene and rDNA internal transcribed spacer ITS1-4 genomic region. Lane 1, 2 negative control of each primer pair (no DNA added); lanes 3, 4 correspond to gene 18S rRNA 1500 pb amplified using primers 18S F and 18S R; lanes 5, 6, 7 and 8, a 600 pb band corresponding to ITS1-4 amplicon using universal primers ITS 1 and ITS 4. MW molecular weight 1 Kb Plus (Invitrogen). The annealing temperature in both cases was 60 °C.
