# Supplementary material for: Generation of BSA-capsaicin Nanoparticles and Their Hormesis Effect on the Rhodotorula mucilaginosa Yeast
Source: Molecules. 2019 Aug 1;24(15):2800. doi: 10.3390/molecules24152800 (PMC6695916; doi:10.3390/molecules24152800)
Supplement: Supplementary file 1 [file molecules-24-02800-s001.pdf]

Supplementary Materials

Generation of BSA-capsaicin Nanoparticles and Their Hormesis Effect on the *Rhodotorula mucilaginosa* Yeast

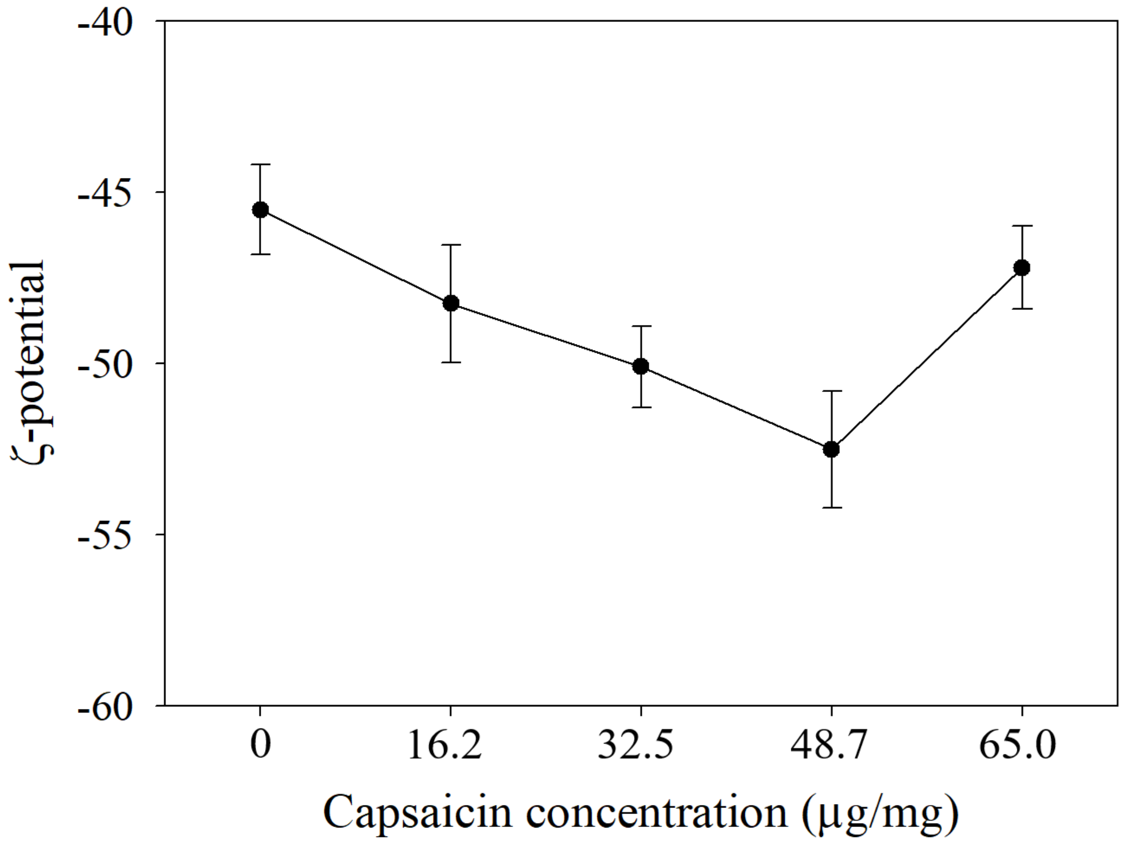

Figure S1: The ζ-potential of BSA-capsaicin nanoparticles showed a decrease in the electronegativity associated to the accumulation of capsaicin into the nanoparticle core. At 65.0 μg/mg the saturation with capsaicin produces a significant increment of the ζ-potential.

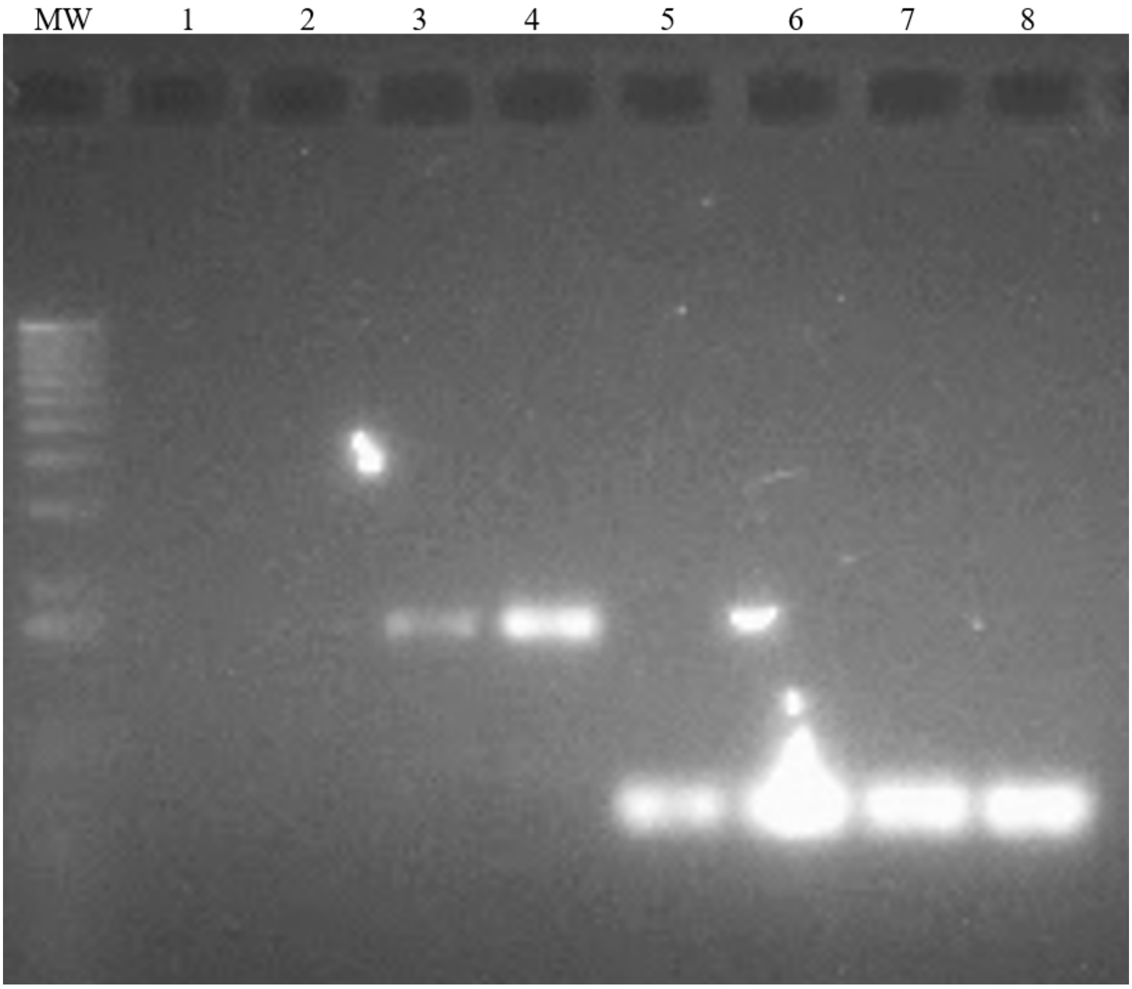

Figure S2: Electrophoretic patterns of PCR products of the 18S rRNA gene and rDNA internal transcribed spacer ITS1-4 genomic region. Lane 1, 2 negative control of each primer pair (no DNA added); lanes 3, 4 correspond to gene 18S rRNA 1500 pb amplified using primers 18S F and 18S R; lanes 5, 6, 7 and 8, a 600 pb band corresponding to ITS1-4 amplicon using universal primers ITS 1 and ITS 4. MW molecular weight 1 Kb Plus (Invitrogen). The annealing temperature in both cases was 60 °C.
